# Interface Management between General Practitioners and Rheumatologists—Results of a Survey Defining a Concept for Future Joint Recommendations

**DOI:** 10.1371/journal.pone.0146149

**Published:** 2016-01-07

**Authors:** Rudolf Puchner, Michael Edlinger, Erich Mur, Gabriele Eberl, Manfred Herold, Peter Kufner, Antonia Puchner, Stephan E. Puchner, Kurt Redlich, Alois Alkin, Klaus Machold

**Affiliations:** 1 Rheumatology Practise Dr Puchner, Wels, Austria; 2 Department of Medical Statistics, Informatics and Health Economics, Medical University of Innsbruck, Innsbruck, Austria; 3 Department of Internal Medicine I, Medical University of Innsbruck, Innsbruck Austria; 4 Klinikum Malcherhof Baden, Baden bei Wien, Austria; 5 Private Practise Dr Kufner, Innsbruck, Austria; 6 Division of Rheumatology, Department of Internal Medicine III, Medical University of Vienna, Vienna, Austria; 7 Department of Orthopaedic Surgery, Medical University of Vienna, Vienna, Austria; 8 Centre of Excellence in Medicine, Linz, Austria; Oregon Health & Science University, UNITED STATES

## Abstract

**Objective:**

To measure the views of general practitioners (GPs) and rheumatologists in a nationwide evaluation, so as to optimise their cooperation in managing patients with inflammatory rheumatic diseases.

**Methods:**

A questionnaire covering aspects of collaboration was sent, both by mail and/or by email, to all GPs and rheumatologists in Austria. Topics covered were (i) examinations and interventions to be performed before referral, (ii) the spectrum of diseases to be referred, and (iii) the role of GPs in follow-up and continuous management of patients.

**Results:**

1,229 GPs of the 4,016 GPs (31%) and 110 of the 180 rheumatologists (61%) responded to the questionnaire. In cases of suspected arthritis, 99% of the GPs and 92% of the rheumatologists recommended specific laboratory tests, and 92% and 70%, respectively, recommended X-rays of affected joints before referral. Rheumatoid arthritis and spondyloarthritis, psoriatic arthritis and connective tissue disease were unanimously seen as indications for referral to a rheumatologist. Only 12% of rheumatologists felt responsible for the treatment of hand osteoarthritis and fibromyalgia. 80% of GPs and 85% of rheumatologists were of the opinion that treatment with disease-modifying drugs should be initiated by a specialist. Subsequent drug prescription and administration by GPs was supported by a majority of GPs and rheumatologists, with a concomitant rheumatologist follow-up every three to six months.

**Conclusion:**

The considerable consensus between the two professional groups constitutes a solid base for future joint recommendations, with the aim to accelerate the diagnostic process and the initiation of adequate therapy.

## Introduction

Acute and chronic rheumatic diseases, such as osteoarthritis, gout or degenerative spine disease, are frequent and have a profound impact on patients’ and their families’ quality of life [1, 2]. Patients usually suffer from pain and disability to a varying extent. Therefore, a considerable amount of general practitioners' (GPs) working time is devoted to the management of rheumatic diseases [3]. Among conditions with a component of arthritis, such as rheumatoid arthritis (RA), connective tissue diseases (CTDs) or ankylosing spondylitis (AS), which are less common than the degenerative ones, many, such as RA, require rapid referral to a specialist. The reason being that they can inflict damage within a short period of time. In general, a GP is the primary contact for all these patients and they then have to decide whether rheumatological consultation is required, or how rapidly patients should be referred. A delay in the referral to a specialist of a patient with early rheumatoid arthritis increases the danger of joint destruction, with the potential risk of longer sick leave, early disability, or the initiation of pathways to costly surgical intervention later. Indeed, for more than a decade, the development of new drugs has decisively changed and improved the treatment options and outcome of chronic inflammatory rheumatic diseases [4–9]. Whereas, in the 1990s rheumatologists were content with somewhat reducing the swollen joint count using disease modifying, anti-rheumatic drugs (DMARDs), nowadays a “treat-to-target” approach is preferred with the aim of achieving remission, or at least low disease activity [10] and today the respective therapeutic means are available.

On the other hand, the unnecessary referral of patients with a rheumatic condition to a rheumatologist is the most important reason for the increasing work overload in rheumatology practices, distracting them from patients with more complex rheumatic problems such as acute and chronic inflammatory diseases [3]. Therefore, communication and information exchange between rheumatologists and GPs needs to be improved, and to this end, referral recommendations were already developed many years ago, at least for RA and AS [11, 12]. This was done in the hope of reducing waiting times in rheumatology outpatient clinics and practices by a better selection of patients, earlier diagnosis and earlier implementation of therapy, thereby resulting in better patient outcome.

The primary-specialist care interface is a key organisational feature of many health care systems. Some countries have a similar formal referral system, for example, the UK, the Netherlands and Denmark, where primary care physicians provide health care and act as gatekeepers with responsibility for defining which patients require specialist care. Other countries have a less formalised referral system, for example, Germany, Switzerland and Austria, where patients have free choice of doctor, whether primary care physician or specialist [13].

Overall, delays in consulting a GP, delays in referral to a rheumatologist, waiting times for appointments and delays in correct diagnosis by a rheumatologist after referral, are the main obstacles to rapid initiation of appropriate therapy.

However, even with the application of appropriate referral recommendations, the delay in the diagnostic process with the rheumatologist still might not be reduced. This delay could be decreased if the patient is referred together with appropriate test results, whether laboratory or imaging. Therefore, it may be important to decide which tests or procedures would help to improve diagnostic accuracy and avoid inappropriate testing or referrals. This latter aspect was addressed in this study among GPs and rheumatologists, whose opinions regarding appropriate testing before referral were assessed using a questionnaire survey. Topics included requests for laboratory tests, investigations and follow-up examinations performed by GPs, as well as listing the patients with a defined spectrum of diseases who should be referred to the specialist.

Our aim was to compare results of both groups of physicians in order to find possibilities for improving the efficiency of this cooperation between GPs and rheumatologists.

## Materials and Methods

In cooperation with the board of the Austrian Society of Rheumatology, a questionnaire was designed to address various aspects of cooperation between rheumatologists and GPs. A pilot phase was carried out in which 46 out of 98 invited rheumatologists participated. The pilot and final questionnaires differed only slightly in wording. In a second phase, 100 GPs in training practices were interviewed. Additionally the understanding of the meaning of “a clinical suspicion of Lyme disease”, of “antibodies against citrullinated peptides (ACPA)” and “inflammatory back pain” was tested in a smaller sample of GPs, with results indicating sufficient knowledge.

The final questionnaire was the result of expert evaluation by board member rheumatologists, a GP, an epidemiologist, and a sociologist. The form was almost identical for both GPs and rheumatologists, except for three questions about professional experience in the treatment of rheumatic diseases which was only for GPs.

Questions addressed the following issues:

- The laboratory tests and radiological examinations that should be ordered by GPs before referral to a rheumatologist, in patients with arthritis or suspicion of inflammatory back pain.
- The rheumatic diseases that should be presented to a rheumatologist and how their referral should be organised.
- The interventions that should be performed by GPs in patients with inflammatory rheumatic diseases before referral to a rheumatologist and afterwards during the long-term treatment of the disease.

The questionnaire, with its covering letter, was sent out to all 4,016 Austrian GPs and to all 180 Austrian internists with a rheumatology subspecialty. We deliberately excluded retired rheumatologists and those with formal qualification but no current work as a rheumatologist. The participants were given a five week deadline to return the questionnaire; a reminder was sent after three weeks. The survey period was from the 12^th^ of June to the 15^th^ of July 2013. The questionnaire could be filled out either online or returned by regular mail.

The survey was a project of the Austrian Society of Rheumatology and was supported by the Austrian Medical Association and the Austrian Society for General Practice and Family Medicine.

Fisher's exact test or χ^2^-test for trend was used to statistically evaluate differences between the two professional groups. The data were analysed with SPSS version 19.

In our study an ethics committee was not necessary. There was no research in humans. No patient data was obtained in this questionnaire. No patients were involved in this study.

This questionnaire survey exclusively asked for health care data. No questions referred to any personal data regarding patient’s personal medical history were asked. General practitioners and rheumatologists were asked for their professional opinions related to health care management.

All practising General practitioners and rheumatologists in Austria, all of them are registered in the Austrian Medical Association were invited to participate in this survey.

The authors of the manuscript had no access to identifying participant information at any time.

## Results

1,229 of the 4,016 GPs (31%) and 110 of the 180 rheumatologists (61%) sent back evaluable questionnaires (S1 Dataset). Demographic data and other characteristics such as an urban or rural area of workplace are given in Tables 1, 2 and 3. Most GPs see 3 to 6 patients with inflammatory rheumatic diseases in one month. Asked about their competence in its treatment, the majority of GPs rated themselves at a minimum of 3 on a scale of 1 to 6; 1 denoting very competent and 6 not at all. For over 70% of GPs the distance from their practice to the nearest rheumatologist was less than 30 km (Table 2).The majority of rheumatologists are hospital based (Table 3).

**Table 1:** Characteristics of the physicians who responded to the survey.

	General practitioners n (%)	Rheumatologists n (%)
Participation	1229 (31%)	110 (61%)
Age (years), median (IQR)	55 (49; 59)	50 (43; 54)
No answer	42 (3%)	1 (1%)
Sex		
Male	878 (71%)	84 (76%)
No answer	3 (0%)	1 (1%)
Population of municipality where workplace is situated		
- 10,000	734 (60%)	17 (15%)
10,001–100,000	217 (18%)	34 (31%)
100,001 +	196 (16%)	49 (45%)
No answer	82 (7%)	10 (9%)

Abbreviation: IQR = interquartile range.

**Table 2:** GPs’ characteristics.

Distance from GP practice to rheumatologist	
≤ 10 km.	459 (37%)
11–30 km.	412 (34%)
31–50 km.	194 (16%)
51–100 km.	87 (7%)
≥ 101 + km.	19 (2%)
No answer	58 (5%)
Number of patients with inflammatory rheumatic diseases per month	
none	2 (0%)
1 or 2	91 (7%)
3 or 4	310 (25%)
5 or 6	310 (25%)
7 or 8	155 (13%)
≥ 9	303 (25%)
No answer	58 (5%)
Most recent further rheumatological education	
before 2010	194 (16%)
2010	70 (6%)
2011	172 (14%)
2012	500 (41%)
2013	226 (18%)
No answer	67 (5%)
Experience in the treatment of rheumatic diseases	
1 (a great deal)	43 (3%)
2	290 (24%)
3	579 (47%)
4	220 (18%)
5	46 (4%)
6 (none at all)	2 (0%)
No answer	49 (4%)

**Table 3:** Rheumatologists’ characteristics.

Work situation	
Rheumatologist in private practise	30 (27%)
Rheumatologist in non-university hospital medical dept.	23 (21%)
Rheumatologist at university hospital rheumatology dept.	50 (45%)
Other	2 (2%)
No answer	5 (5%)
Number of patients with inflammatory rheumatic diseases per month	
≤ 50	39 (35%)
51–100	29 (26%)
101–150	18 (16%)
≥ 151	18 (16%)
No answer	6 (5%)

### Pre-referral tests recommended for cases of arthritis or suspected, inflammatory back pain

**Fig 1:**
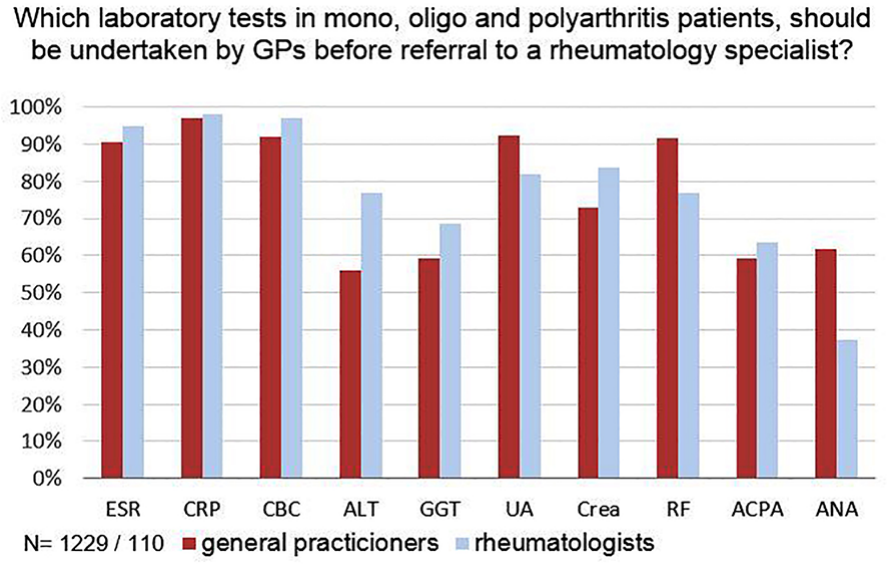
Opinion on laboratory examinations to be performed by the GP prior to referral to the rheumatologist in case of arthritis. Abbreviations: ESR, erythrocyte sedimentation rate; CRP, C-reactive protein; CBC, complete blood count; ALT, alanine transaminase; GGT, gamma-glutamyl transferase; UA, uric acid; Crea, creatinine; RF, rheumatoid factor; ACPA, antibodies against citrullinated protein/peptide antigens; ANA, anti-nuclear-antibodies. The differences between GPs and rheumatologists were statistically significant for ALT (p<0.001), Uric acid (p = 0.001), Creatinine (p = 0.035), RF (p<0.001), ANA (p<0.001). * p value based on Fisher's exact test.

In cases of monoarthritis, oligoarthritis, or polyarthritis 1,215 (100%) of the GPs and 101 (92%) of the rheumatologists recommended laboratory testing before referral to a rheumatologist. There were similar results among all participants calling for tests for acute phase parameters and complete blood count, before referral. Fewer rheumatologists than GPs recommended rheumatoid factor testing (77% vs 91%, p<0.001), a small majority of GPs (59%) and rheumatologists (62%) voted for testing for antibodies against citrullinated peptides (Fig 1). Few GPs recommended routine Borrelia serology, although they did so significantly more frequently than rheumatologists (17% vs. 2% p<0.001). In cases of a substantiated clinical suspicion of Lyme disease however, 77% of GPs and 57% of rheumatologists said they would prefer Borrelia serology determined in advance.

Conventional radiological imaging of the affected joints in cases of arthritis before referral was recommended by 94% of the GPs and 71% of the rheumatologists (p<0.001).

**Fig 2:**
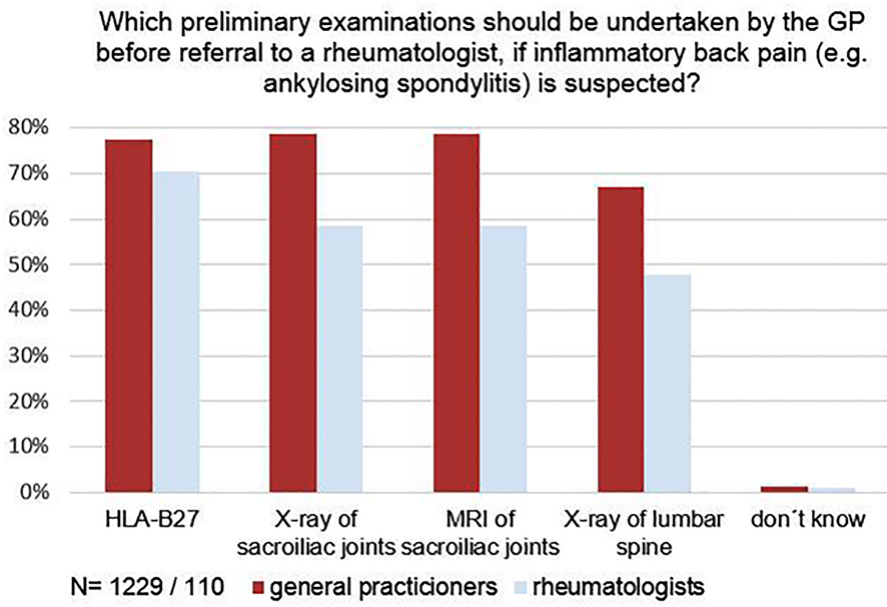
Opinion on necessity of examinations to be acquired by the GP before referral to a rheumatologist, if inflammatory back pain is suspected. The difference between GPs and rheumatologists was statistically significant for x-ray of sacroiliac joints and x-ray of lumbar spine (both p<0.001). * p value based on Fisher's exact test

In patients with suspected inflammatory back pain, again, a majority of both GPs and rheumatologists wanted HLA-B27 tested, prior to referral to the specialist. 81% of the GPs and 63% of the rheumatologists also requested a conventional x-ray of the sacroiliac joints and 69% and 51% respectively, a conventional X-ray of the lumbar spine. 58% of the GPs and 44% of the rheumatologists asked for both, and 30% and 18%, respectively, required just one of these (out of these, 70% of the GPs and 80% of the rheumatologists wanted an x-ray of the sacroiliac joints). In both groups, 25% recommended magnetic resonance imaging (MRI) prior to referral to the rheumatologist (Fig 2).

### Diseases to be referred to a rheumatologist (in addition to monoarthritis, oligoarthritis and polyarthritis, or suspicion of inflammatory back pain)

The majority of responding GPs (78%) and rheumatologists (90%) stated that patients with connective tissue disease should always be referred to a rheumatologist, even in the absence of joint involvement. In addition, the respondents felt that patients with psoriatic arthritis should be presented to a specialist in case of joint involvement (81% and 82% respectively), and 18% of rheumatologists also wanted to concern themselves with those psoriasis patients not displaying joint involvement, where 12% of the GPs felt that was necessary.

Only 11% of the GPs and 12% of the rheumatologists thought that patients with osteoarthritis of the hands should always see a rheumatologist, while 65% and 77% respectively, recommended presentation of these patients for differential diagnosis in case of active/inflammatory arthritis. With regard to suspected fibromyalgia, 89% of GPs would refer these patients: 35% always and 54% of them only to rule out inflammatory rheumatic disease. Among the rheumatologists 12% always felt responsible for the treatment of such patients, while 74% were of the opinion that patients with fibromyalgia should only present to a rheumatologist to rule out inflammatory rheumatic disease. In the case of polymyalgia rheumatica, 53% of the GPs and 84% of the rheumatologists suggested consistent referral of suspected cases to the specialist, another 45% of the GPs would refer only if atypical.

### Interventions by GPs in patients with inflammatory rheumatic diseases before referral to a rheumatologist

GPs and rheumatologists were asked if GPs should start glucocorticoid (GC) treatment in patients that had shown an insufficient response to non-steroidal anti-inflammatory drugs (NSAIDs). There was significant disagreement between both groups (Table 4). Whereas 43% of GPs would always prescribe GC, among the rheumatologists only 11% were of the opinion that they should be prescribed by the GP before referral at all (p<0.001) and 32% even discouraged their prescription to avoid masking of disease symptoms. There were significant differences in varying demographics of GPs. In urban areas (> 10000 inhabitants) GPs tend towards saying “no” (chi-squared test for trend, p = 0, 01). GPs with more self-declared experience in the field of musculoskeletal disorders (group 1 and 2 see Table 3) have a strong tendency to say “yes” (chi-squared test for trend, p<0,001).

**Table 4:** Opinion of general practitioners (n = 1229) and rheumatologists (n = 110) on interventions by general practitioners in patients with inflammatory rheumatic diseases.

	General practitioners	Rheumatologists	p value *
Therapy with glucocorticoids by the GP before referral			
no	98 (8%)	35 (32%)	
sometimes ^a^	583 (49%)	62 (57%)	
yes, always	512 (43%)	12 (11%)	<0.001
Therapy with DMARDs by the GP before referral			
no	553 (46%)	52 (47%)	
sometimes ^a^	401 (34%)	41 (37%)	
yes, always	243 (20%)	17 (15%)	0.440
Clinical check-up and laboratory tests during treatment with DMARDs by the GP			
no	4 (0%)	0 (0%)	
in exceptional cases	42 (4%)	6 (5%)	
yes, but specialist check up every 6 mo.	559 (47%)	32 (29%)	
yes, but specialist check-up every 3 mo.	225 (19%)	63 (57%)	
yes, only by the GP	369 (31%)	9 (8%)	0.387
Administration of biologics performed by the GP			
no	222 (22%)	15 (14%)	
yes	790 (78%)	91 (86%)	0.062
Subsequent prescription of biologics by the GP			
no	108 (9%)	28 (26%)	
yes	1040 (91%)	81 (74%)	<0.001

^a^ Restricted to special circumstances (e.g. extraordinarily long waiting times for the rheumatology appointment or limited availability of a rheumatologist)

* p value based on χ^2^ for trend or Fisher's exact test

GPs and rheumatologists generally agreed that initiation of therapy with synthetic disease modifying drugs (sDMARDs) should only be done by a specialist for rheumatic diseases and a third of both groups also agreed that in special circumstances, such as the limited availability of a rheumatologist, sDMARD therapy could also be started by a GP.

For patients with stable disease and/or on stable treatment, the majority of the rheumatologists questioned believed that clinical check-ups and lab results should be the responsibility of the GP, but that specialist appointments should take place every three to six months. Also, a majority of the GPs were of the same opinion, although roughly one third of them believed that clinical check-ups and lab results should be the sole responsibility of the GP.

GPs and rheumatologists agreed that the administration of biologic DMARDs (bDMARDs) could also be performed by GPs. Regarding follow-up prescriptions of bDMARDs, significantly fewer rheumatologists than GPs thought that this could be left to the GP (p<0.001) (Table 4).

The respondents´ opinions were markedly split regarding their preferred courses of action in cases of worsening inflammatory joint disease and/or treatment side-effects. Only 12% of the GPs, but 51% of the rheumatologists, would opt for immediate presentation to a specialist in these cases, the rest would leave the initial examination to the GP (88% and 49% respectively p<0.001). In cases of side-effects of treatment, 78% of the GPs and 48% of the rheumatologists believed that the GP should be visited first; 22% and 52% respectively, believed that the specialist should be involved immediately (p<0.001). In urban areas the majority of the GPs voted for immediate contact with the specialist (Fisher's exact test, p<0,001). GPs with more self-declared experience prefer to see those patients first in their practice (Fisher's exact test p = 0, 03).

### Urgent referrals

GPs and rheumatologists were asked about correct procedure in cases of urgent referral. 69% of GPs and 87% of rheumatologists preferred telephone contact by the GP. To simplify the recognition of urgent patients the word “urgent” on the referral letter was appreciated by 40% of GPs and 20% of rheumatologists; a service telephone at rheumatology outpatient departments by 37% and 22%; and email contact with guaranteed response within a certain time limit, by 14% and 18% respectively (multiple answers were possible).

## Discussion

In the present study we surveyed practising GPs and rheumatologists in Austria for their opinions and recommendations regarding their respective roles in the management of patients with rheumatic problems. The results demonstrate that a vast majority, of both GPs and rheumatologists, prefer to have some laboratory results and radiological examinations of affected joints available before first consultation of a specialist, in order to allow acceleration of the diagnostic process and initiation of treatment. With regard to the spectrum of diseases, the majority of respondents stated that in addition to monoarthritis, oligoarthritis and polyarthritis (or suspicion of inflammatory back pain), patients with connective tissue diseases should definitely be seen by a rheumatologist.

Only a minority of the surveyed rheumatologists regarded themselves as the primary referral address for patients who have osteoarthritis and fibromyalgia with the exception of cases in which an inflammatory rheumatic disease has to be excluded. Regarding fibromyalgia, this opinion is supported by a Dutch study showing that fibromyalgia can also be diagnosed and adequately treated by a skilled GP [7]. The implication of these answers might be a shared care through GPs and rheumatologists. The GP is the first and most important contact person and a gatekeeper, while the rheumatologist rules out an inflammatory rheumatic disease and gives support in pain management. However, the roles of psychiatrists, pain specialists, physical medicine specialists and non-physician clinicians who might also be involved in fibromyalgia care was not a distinct topic of the survey.

The discordant view about GC usage needs further discussion. The start of GC treatment by a GP before referral, especially in early arthritis patients, could mask symptoms and therefore delay diagnosis. It should be reserved for emergency situations such as the severe manifestation of symptoms or unduly long waiting times for a specialist appointment. Future training and the introduction of guidelines for appropriate use of GC by GPs might be of help. Likewise, follow-up prescription of bDMARDs by GPs which is supported by a majority of rheumatologists but is seen feasible by significantly more GPs, may benefit from clear guidelines. Clinical and laboratory monitoring of DMARDs as well as the administration of bDMARDs (with the exception of rituximab) by GPs is seen as feasible. Because the latter appears safe [14, 15] this may help economise on the care of patients with rheumatic diseases further. Supervision of treatment with DMARDs may also be executed by GPs, with three to six-monthly specialist appointments in order to optimise patient`s security.

Referral recommendations, at least for RA and AS, are well established and were published some years ago [11, 12] as a tool for primary care doctors to identify potential patients with active disease. The aim of our study was to facilitate interface management between primary and secondary care.

According to the literature, GP’s in Austria and Germany see an average of 0.4 incident RA patients per year—not only is RA hard to diagnose, it is also relatively rare in a GP’s office compared to, e.g., cardiovascular diseases. [16,17].

In a severe disease like RA it seems to be more important to upgrade interface management than to put too much effort in GPs’ training.

The major limitation of this study is its restriction to the Austrian health care system. In this system, in a country with universal health insurance coverage, the GPs role is quite extensive and the burden of caring for a multitude of medical complaints restricts their ability to deal with more complex cases. Whereas this is probably also true in many other countries, the system currently assigns no role to other professions such as specialist nurses or therapists to relieve or to assist the GP in management of e.g. referrals, follow-up or repeat prescriptions. This may have influenced some of the responses in this study.

Another limitation concerns the response rate, even though that of the rheumatologists was quite high. Less than a third (31%) of all GPs actually decided to participate in this study. However, this is a well-known problem with GP research and especially in large surveys with some thousand participants, a response rate between 30–34% is commonly seen and only achievable with reminders and public support [18,19]. A relatively large number of GPs from Upper Austria responded, a few from the capital Vienna, and there was also an under-representation of GPs from the major cities with over 100,000 inhabitants. This might be due to more differentiation/specialisation of doctors in urban areas and therefore generally less contact of GPs with rheumatic patients, thus leading to less motivation to participate. In more rural areas the opposite is true with regard to specialisation and therefore GP participation might have been higher due to this. In addition, we do not know about the experience of the non-participants in caring for rheumatic diseases/patients. It appears possible that the respondents were more interested in rheumatological problems and therefore more motivated to answer the survey.

**Fig 3:**
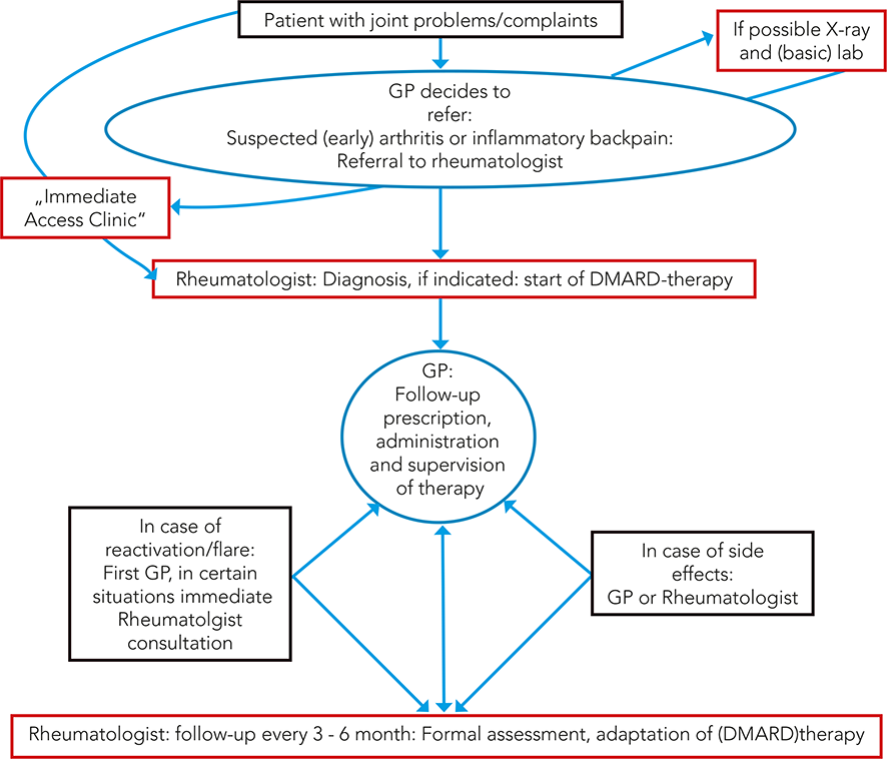
Optimised management of RA.

Taken together, the results of our survey demonstrate the necessity for clearly defined arrangements for optimised co-operation between GPs and rheumatologists (Fig 3). In many areas a high degree of agreement between the surveyed GPs and rheumatologists was found, in particular with regard to the recommended pre-referral tests. The important role of the GP in follow-up and surveillance of the patients is acknowledged by the majority of both professions. The management of patients in cases of disease flare or drug side effects needs further discussion. The possibility of rapid access rheumatology consultation could facilitate and accelerate treatment in these situations [20].

A shared responsibility of both specialists and primary care physicians for treating patients with inflammatory rheumatic diseases is repeatedly discussed and called for in the literature [21–24]. We carried out a nationwide evaluation on the views of primary care providers and rheumatologists, as far as we know the first one ever done up to now, in order to improve chronic disease care. There is considerable agreement between the two professional groups and such consensus constitutes a solid base onto which joint recommendations can be formulated. Such joint recommendations, developed by all professionals concerned, will potentially find more acceptance and could lead to optimisation of care.

This would probably be useful for (i) reducing the time to diagnosis and initiation of therapy for patients with RA and other inflammatory rheumatic diseases, (ii) improving cooperative care between GPs and rheumatologists after diagnoses and treatments have been established, and (iii) last but not least, lowering overall costs.

## Supporting Information

S1 Dataset (XLSX)
